# Correlation Between Site and Stage of Breast Cancer in Women

**DOI:** 10.7759/cureus.22672

**Published:** 2022-02-27

**Authors:** Aamera Shah, Ghulam Haider, Nargis Abro, Sana Hashmat, Sanam Chandio, Abdulla Shaikh, Kiran Abbas

**Affiliations:** 1 Department of Oncology, Jinnah Postgraduate Medical Centre, Karachi, PAK

**Keywords:** younger age, upper outer quadrant, stage of tumor, site of tumor, early stage, breast cancer, advanced stage

## Abstract

Introduction

Breast cancer is a worldwide public health issue and a primary cause of death among women. The present study aimed to assess the correlation between site and stage of breast cancer with respect to age among females.

Methods

A prospective observational study was conducted at the Medical Oncology Department, Jinnah Postgraduate Medical Centre, Karachi, Pakistan from May 2020 to June 2021. Female patients of 18 years or older with a confirmed diagnosis of breast cancer were included in the study. Histopathological reports were evaluated for tumor characteristics such as histological type, laterality, location, tumor size, grade, lymph node status, and stage of the tumor.

Results

The majority of the patients presented with advanced stages of tumor. Among all stages of breast tumor, the upper outer quadrant was the most frequent location of the tumor. The majority of the patients with cancer in the upper inner quadrant were diagnosed with stage I (28.57%) (p = 0.011). In contrast, the majority of the upper outer quadrant lesions were identified as stage III and stage IV (p < 0.0001). In patients of age <40 years, statistically significant differences in proportions of tumor location with respect to the stage of the tumor were observed (p = 0.018).

Conclusion

The upper outer quadrant and stage III of tumor are the most common site and stage of breast tumor in our population. There is a significant relationship between site and stage of breast tumor. Younger-aged patients had a significantly higher rate of cancers located in the upper outer quadrant in advance stages as compared to other quadrants.

## Introduction

According to the World Health Organization, about 2.3 million females were diagnosed with breast cancer in 2020, with 685,000 deaths worldwide [1]. Breast cancer is becoming more common in both developing and developed countries; however, due to early detection and treatment, developed countries have higher survival and prognosis than developing countries [2].

Pakistan has Asia's highest age-standardized incidence rate of breast cancer, i.e., 2.5 times than that of India and Iran [3]. Pakistan is ranked 10th in the world for breast cancer incidence, with a 27% estimated death rate [4]. The majority of breast cancer cases are detected at an advanced stage (stages III and IV), and the percentage of patients diagnosed at an early stage (stages I and II) in Pakistan has been stable over the last two decades [5].

Location or site of tumor within the breast has been identified as an independent risk factor for the poor prognosis. For example, the upper inner quadrant had a much lower rate of axillary lymph node metastasis than the other quadrants (21% vs. 33%). Tumors in the upper outer quadrant, on the other hand, are the most prevalent and have been linked to a higher rate of survival when compared to other quadrants; survival data for non-upper outer quadrant tumors have been mixed, with findings indicating reduced survival in the lower inner quadrant, as well as periareolar, medial, or lower regions [6]. Whereas other researchers have found no significant relationship between tumor site and outcome [7]. In Pakistan, left-sided breast cancer was much more common than right-sided breast cancer. Although the cause of the increased occurrence of left-sided breast cancer is unknown, several researchers have suggested a possible link with substantially bigger left breast size, diagnostic bias owing to dominant right-handedness, unilateral lactation, or more dense left breast [8].

The rationale behind the study was that as in our country, Pakistan, we do not have a central registry where all cancer cases are reported, therefore, we do not have much knowledge on the epidemiological characteristics of breast cancer; however, Western studies have shown a rising trend in cases [1]. Furthermore, there is a huge literature gap on the possible correlation between the location of the tumor and the stage of the carcinoma. The goal of this study was to assess if there is a link between breast cancer stage and location with respect to age in females who present to a tertiary care hospital in Karachi, Pakistan. This research would aid in determining whether the tumor site is linked to the breast cancer stage.

## Materials and methods

A prospective, observational study was conducted at the Department of Medical Oncology, Jinnah Postgraduate Medical Centre, Karachi, Pakistan between May 2020 and June 2021. The study was approved by the ethical committee of Jinnah Postgraduate Medical Centre, Karachi (# F.2-81/2021-GENL/61342/JPMC). Written informed consent was obtained from all the eligible participants after narrating the aims and objectives of the study. A non-probability convenience sampling technique was applied for the recruitment of study participants. The sample size was estimated using the proportion of central quadrants as 25% among advanced stages of tumor [6], a margin of error as 5%, and 95% confidence level and the estimated sample size came out as 290 patients. Female patients of 18 years or older with a confirmed diagnosis of breast cancer were included in the study. All cases were diagnosed after histopathological evaluation and comprehensive clinical examination by a consultant with more than 10 years of experience. All patients younger than 18 years, with an inconclusive diagnosis of breast cancer, or non-malignant lesions such as fibroadenoma, and those who had a previous history of mental illness were excluded from the study.

A pre-designed proforma was used to collect data regarding socio-demographic determinants including age, marital status, parity, family history of cancer, smoking history, comorbidities, and residence status. Histopathological reports of all the patients were evaluated for tumor characteristics such as histological type, tumor size, grade, lymph node status, tumor location, and stage of the tumor. All collected data were kept secure and used for research purposes only. No personal identifiers for any patient were recorded. Anonymity was ensured for all patients.

Data were analyzed using Statistical Package for the Social Sciences (SPSS) version 23 (IBM Corp., Armonk, NY). Mean and standard deviations were computed for numeric variables like age at presentation and age at menarche, whereas frequency and percentage were computed for categorical variables like socio-demographic factors and histopathological characteristics. Correlation between the site and stage of tumor was assessed using the chi-square test/Fisher's exact test. Stratified analysis was performed for age. Post-stratification, the association between the site of the tumor and the stage of the tumor was assessed using the chi-square test. A p-value ≤ 0.05 was considered statistically significant.

## Results

A total of 290 patients with a mean age of 46.33 ± 11.17 years (range: 22-80 years) were assessed. The mean at menarche was reported as 12.11 ± 0.92 years (range: 10-14 years). The majority of the females were married (n = 262, 90.3%) and among married females, 90.8% of females had a history of ever breastfeeding (Table 1).

**Table 1:** Baseline characteristics of study variables (n = 290).

Variable	Mean ± SD
Age at presentation (years)	46.33 ± 11.17
Age at menarche (years)	12.11 ± 0.92
Residence	
Rural	72 (24.8%)
Urban	218 (75.2%)
Ethnicity	
Urdu	140 (48.3%)
Sindhi	54 (18.6%)
Punjabi	47 (16.2%)
Pashto	22 (7.6%)
Balochi	22 (7.6%)
Other	5 (1.7%)
Education	
Illiterate	170 (58.6%)
Primary	84 (29%)
Matric	18 (6.2%)
Intermediate	9 (3.1%)
Graduate	7 (2.4%)
Postgraduate	2 (0.7%)
Employment status	
Unemployed	269 (92.8%)
Employed	21 (7.2%)
Marital status	
Married	262 (90.3%)
Unmarried	28 (9.7%)
History of ever breastfeeding	
Yes	238 (82.1%)
No	52 (17.9%)
Family history of cancer	
Yes	52 (17.9%)
No	238 (82.1%)

Most of the females presented with right-sided breast cancer (n = 156, 53.8%), and the upper outer quadrant was the most frequent tumor location (n = 203, 70%). Other tumor characteristics are mentioned in Table 2. Majority of the patients presented with advanced stages of tumor (stages III and IV).

**Table 2:** Clinical characteristics of study participants (n = 290).

Variable	n (%)
Laterality	
Left	129 (44.5%)
Right	156 (53.8%)
Bilateral	5 (1.7%)
Location	
Upper inner quadrant	30 (10.3%)
Lower inner quadrant	41 (14.1%)
Upper outer quadrant	203 (70%)
Lower outer quadrant	13 (4.5%)
Central	3 (1%)
Tumor size	
0-2 cm	22 (7.6%)
2-5 cm	222 (76.6%)
>5 cm	46 (15.9%)
Grade of tumor	
Well-differentiated	13 (4.5%)
Moderately differentiated	191 (65.9%)
Poorly differentiated	86 (29.7%)
Lymph node status	
Positive	237 (81.7%)
Negative	53 (18.3%)
Stage of tumor	
I	7 (2.4%)
II	61 (21%)
III	160 (55.2%)
IV‎	62 (21.4%)
Histopathological type of breast cancer	
Infiltrating lobular carcinoma	21 (7.2%)
Infiltrating ductal carcinoma	266 (91.7%)
Medullary carcinoma	3 (1%)

Among all stages of breast tumor, the upper outer quadrant was the most frequent location of the tumor. Table 3 illustrates the link between each site of the tumor as identified in our study with the stage of the disease. The majority of the patients with cancer in the upper inner quadrant were diagnosed with stage I (28.57%) (p = 0.011). In contrast, the majority of the upper outer quadrant lesions were identified as stage III and stage IV (p < 0.0001).

**Table 3:** Correlation analysis of tumor site and stage among females with breast cancer.

Tumor location	Stage of tumor				P-value
	I (n = 7)	II (n = 61)	III (n = 160)	IV‎ (n = 62)	
Upper inner quadrant					0.011
Yes	2 (28.57%)	12 (19.67%)	13 (8.13%)	3 (4.84%)	
No	5 (71.43%)	49(80.33%)	147 (91.88%)	59 (95.16%)	
Lower inner quadrant					0.172
Yes	1 (14.29%)	14 (22.95%)	19 (11.88%)	7 (11.29%)	
No	6 (85.71%)	47 (77.05%)	141 (88.13%)	55 (88.71%)	
Upper outer quadrant					0.0001
Yes	4 (57.14%)	33 (54.10%)	119 (74.38%)	47 (75.81%)	
No	3 (42.86%)	28 (45.90%)	41 (25.63%)	15 (24.19%)	
Lower outer quadrant					0.777
Yes	0	2 (3.28%)	7 (4.38%)	4 (6.45%)	
No	7 (100%)	59 (96.72%)	153 (95.63%)	58 (93.55%)	
Central					0.805
Yes	0	0	2 (1.25%)	1 (1.61%)	
No	7 (100%)	61 (100%)	158 (98.75%)	61 (98.39%	

The data were stratified into age groups to assess the effect of age on outcome. In the age group of <40 years, the upper outer quadrant was the most frequent tumor location and stage III was the most frequent stage of the tumor. Further, none of the patients had tumors located at the central site. There were statistically significant differences in proportions of tumor location with respect to the stage of tumor in patients of age <40 years (p = 0.018). There were no statistically significant differences in proportions of tumor location with respect to the stage of tumor in patients of age ≥40 years (p = 0.552) (Table 4).

**Table 4:** Stratified analysis with respect to age for correlation between tumor location and stage of tumor among females with breast cancer (n = 290). * P-value < 0.001.

Age groups	Tumor location	Stage of tumor				P-value
		I	II	III	IV‎	
<40 years	Upper inner quadrant	2 (66.7%)	4 (28.6%)	3 (6.4%)	1 (4.8%)	0.018*
	Lower inner quadrant	0	5 (35.7%)	6 (12.8%)	3 (14.3%)	
	Upper outer quadrant	1 (33.3%)	5 (35.7%)	35 (74.5%)	15 (71.4%)	
	Lower outer quadrant	0	0	3 (6.4%)	2 (9.5%)	
	Central	0	0	0	0	
≥40 years	Upper inner quadrant	0	8 (17%)	10 (8.8%)	2 (4.9%)	0.552
	Lower inner quadrant	1 (25%)	9 (19.1%)	13 (11.5%)	4 (9.8%)	
	Upper outer quadrant	3 (75%)	28 (59.6%)	84 (74.3%)	32 (78%)	
	Lower outer quadrant	0	2 (4.3%)	4 (3.5%)	2 (4.9%)	
	Central	0	0	2 (1.8%)	1 (2.4%)	

## Discussion

Breast malignancy is a complex disease with a variety of subtypes and clinical outcomes [9], and the effects of tumor site on tumor stage have not been well investigated in our Pakistani population. The current study evaluated the relationship between breast cancer stage and tumor site to distinguish these individuals and assess prognosis. Further, we also assessed the impact of age on the clinical course of breast cancer.

We found that the majority of the patients were over 40 years old, with a mean age of 46.33 ± 11.17 years. One local study by Akbar et al. found almost identical results, with the average age of females with breast cancer being 47.55 years [10]. Another research by Zeeshan et al. found that the median age was slightly higher at 50 years and that the majority of the females at the time of presentation were over 40 years old [11]. This pattern differs from the frequently reported incidence of breast cancer in women over the age of 60 years [10-13]. More younger women are being diagnosed with breast cancer more likely due to the increased screening and advanced diagnostic utilities. Hence, in developing countries like Pakistan, breast cancer screening should begin while women are younger (in their fourth decade). Breast cancer screening at an early stage might improve overall survival and prognosis. It has been estimated that early screening has increased the five-year survival rate to as high as 85% [14].

A similar study conducted on Iranian women suffering from breast cancer showed that >60% of women had a tumor between 2 cm and 5cm in size, while about 80% of the tumors were larger than 2 cm. About 70% of these patients were diagnosed at stage II of the disease, while 25% of the tumors were detected in stage III. The findings implied that most cases present in advanced stages, with much larger tumor sizes. This means that awareness programs must be initiated, which would promote active participation in breast cancer screening. The female population also needs to be educated on the method and importance of self-assessment [15]. Studies have shown that the site and pattern of tumor metastasis are dependent on the biological types of breast tumors. Liede et al. showed that patients with hormone receptor-positive (HR+) tumors are more likely to present with bone metastasis [16]. A similar study found that hormone receptor-negative (HR-)/human epidermal growth factor receptor 2-positive (HER2+) tumors present with metastasis most commonly involving the liver [17].

The site of the tumor is an independent prognostic factor for optimizing treatment and predicting disease outcomes [6,8]. According to the results of the previous study, the upper outer quadrant is the most frequent site of the tumor (52%) as compared to other quadrants. The second most frequent site reported was the upper inner quadrant (16%) [6]. In our study, we also found that the most frequent site of the tumor was the upper outer quadrant, with the lower inner quadrant being the second most common site. Our findings are consistent with the published studies for Asian women, Eastern women, and Western women [1,14,18].

In our study, we discovered a significant relationship between tumor stage and tumor location (p = 0.0001). We revealed that the proportion of upper outer quadrants was higher in all stages of cancer than in other quadrants. Furthermore, we revealed that the proportion of upper outer quadrant and stage III was greater in both age groups (i.e., age < 40 years and age ≥ 40 years), although there was only a significant correlation between site and stage of tumor in the age group of <40 years (p = 0.018). In another study by Rummel et al., dissimilar results have been reported and the proportion of the upper outer quadrant was higher in the early stages (I and II) than in late stages (III and IV). The authors found central quadrants as the most frequent site in late stages as compared to other quadrants. They also concluded that the relationship between site and stage of tumor was statistically significant (p = 0.003) [6]. The increased use of cosmetics applied to the nearby underarm and upper breast region may account for the higher proportion of upper outer quadrants in younger age groups [18,19].

We revealed that most women had right-sided breast cancer, which is in contrast to previously published literature [10-15]. It is speculated that there may be some molecular factors at play here. In conclusion, the current study revealed a correlation between the stage and location of breast cancer along with other significant findings; however, our study was hampered by certain limitations. For instance, the study was conducted in a single center and included an undiversified sample size; thus, limiting the interference of the current findings to the general population. Therefore, large-scale studies are warranted in the future to explore the research subject more extensively.

## Conclusions

The present study indicates that there is a significant relationship between tumor stage and tumor location. The frequency of upper outer quadrants was significantly higher in all stages of cancer as compared to other locations. Furthermore, the findings revealed that the occurrence of stage III in upper outer quadrant cancer was statistically significant. In short, the upper outer quadrant and stage III of the tumor are the most common site and stage of breast tumor in our population. Younger age had a significantly higher proportion of upper outer quadrant cancer in advance stages as compared to other quadrants.
